# Examining the Effect of Virtual Learning on Canadian Pre-Clerkship Medical Student Well-Being During the COVID-19 Pandemic

**DOI:** 10.5334/pme.1184

**Published:** 2023-11-02

**Authors:** Nikita Ollen-Bittle, Asaanth Sivajohan, Joshua Jesin, Majid Gasim, Christopher Watling

**Affiliations:** 1 Department of Medicine, Schulich School of Medicine and Dentistry, Western University, London, ON, CA; 2 Department of Anatomy and Cell Biology, Schulich School of Medicine and Dentistry, Western University, London, ON, CA; 3 Centre for Education Research & Innovation, Schulich School of Medicine and Dentistry, Western University, London, ON, CA; 4 Department of Oncology, Schulich School of Medicine and Dentistry, Western University, London, ON, CA

## Abstract

**Introduction:**

The restrictions of the COVID-19 pandemic resulted in the broad and abrupt incorporation of virtual/online learning into medical school curricula. While current literature explores the effectiveness and economic advantages of virtual curricula, robust literature surrounding the effect of virtual learning on medical student well-being is needed. This study aims to explore the effects of a predominantly virtual curriculum on pre-clerkship medical student well-being.

**Methods:**

This study followed a constructivist grounded theory approach. During the 2020-2021 and 2021-2022 academic years, students in pre-clerkship medical studies at Western University in Canada were interviewed by medical student researchers over Zoom. Data was analyzed iteratively using constant comparison.

**Results:**

We found that students experiencing virtual learning faced two key challenges: 1) virtual learning may be associated with an increased sense of social isolation, negatively affecting wellbeing, 2) virtual learning may impede or delay the development of trainees’ professional identity. With time, however, we found that many students were able to adapt by using protective coping strategies that enabled them to appreciate positive elements of online learning, such as its flexibility.

**Discussion:**

When incorporating virtual learning into medical education, curriculum developers should prioritize optimizing existing and creating new ways for students to interact with both peers and faculty to strengthen medical student identity and combat feelings of social isolation.

## Introduction

While online learning has been used in medical education to varying degrees in the past, the COVID-19 pandemic introduced the abrupt and total adoption of online learning by medical schools worldwide [1,2,3]. Virtual/online learning is loosely defined as a learning environment where instructors and students are separated by distance and/or time and facilitate learning through a computer [4,5]. Despite being necessitated by circumstance, virtual learning has received a high degree of institutional attention and has become more commonplace [2,3]. The economic benefits of virtual learning include reduced cost of staffing, transportation and accommodation [4,5]. Furthermore, benefits include catering to individual needs, increased convenience, and facilitation of active learning [3,6]. Despite the increasing prevalence of virtual education, the vast majority of corresponding literature focuses on the economic and educational considerations. Literature extrapolating the relationship between virtual learning and student mental health is scarce and critically needed. Research to date has yielded mixed and somewhat conflicting results: studies have shown that the convenience and flexibility of online learning can lead to reduced levels of stress among some learners [4,7]. Conversely, virtual learning has also been found to be associated with increased levels of isolation, anxiety and depression among students [8,9].

Medical students face significant threats to mental health partly due to stressors associated with the demands of medical education [10,11,12]. It is well established that the challenging academic workload hinders a good work-life balance, while the competitive nature of medical programs, perfectionist type personalities and exposure to criticism predispose medical students to imposter syndrome and burnout [13]. A recent study showed that relative to age-matched postsecondary graduates, Canadian medical students had significantly higher rates of mood disorders, anxiety disorders, suicidal ideation, and psychological distress [14]. Prior to the commencement of clerkship, up to as many as 71% of medical students show signs of burnout [15]. It is therefore imperative that any changes that may drastically change the day-to-day of a medical student education be carefully considered.

Current research suggests that virtual learning can have a negative impact on the perceived mental health of students [16,17,18]. Some of the described findings include an increase in the amount of anxiety, fear and depression among students [17,18]. The lack of social contact, and technical challenges associated with the virtual environment seem to potentiate these issues [19]. While most of these studies have looked at the impact of virtual learning on mental health, few have focused specifically on the medical student population and even fewer have examined this using a qualitative methodology to explore *how* and *why* virtual learning impacts student wellbeing, and how students might adapt over time [20].

Therefore, in this study, we ask how virtual learning affects pre-clerkship medical student wellbeing, and how these impacts evolve over time. This knowledge will allow stakeholders to evaluate virtual learning from a more holistic perspective such that it can be integrated in a way that is mindful of medical student well-being.

## Methods

For this exploratory study, we used constructivist grounded theory (CGT) methodology [21]. The nature of our research question requires both the subjective interpretation of participant experiences and the subsequent co-construction of knowledge between participant and researcher. This is highly emphasized in grounded theory approaches. Furthermore, the inductive nature of grounded theory is helpful when studying these emerging topics since it lends itself to the identification of new patterns and relationships within the data. It is critical to define what “theory” means in the context of our CGT study and we primarily refer to Charmaz’s definition where she states: “Theories offer accounts for what happens, how it ensues, and may aim to account for why it happened.” [21]. In the interpretative research tradition, theory aims to conceptualize a studied process or phenomenon and to understand it in abstract terms.

### Study Design & Context

Students who were in their first or second year of medical studies during the implementation of curricular changes responding to the COVID-19 pandemic (academic years 2020–2021 and 2021–2022), were recruited from the Schulich School of Medicine, Western University, Ontario Canada to discuss their experiences with online learning and its impacts on student mental health. Online learning formats included: i) Recorded faculty delivered lectures designed to be viewed in a flipped-classroom format, ii) real-time case-based group learning with 5–10 students facilitated by a physician faculty member over video conferencing software, iii) real-time sessions focused on applications of recorded content with faculty addressing entire cohort over video conferencing software. During the latter half of the 2021–2022 academic year, many of the type ii and iii learning formats returned to in-person. Any discussion of an online learning or virtual learning curriculum refers to a curricular format where a majority of the educational sessions took place online.

### Recruitment and Consent Procedures

Participation in this study was entirely voluntary and had no bearing on participants’ academic standing. Participants were initially contacted by a member of the Undergraduate Medical Education office via their institutional e-mail with a recruitment e-mail. Interested parties were invited to contact the researchers to obtain a Letter of Information. Parties that received the Letter of Information and wished to participate received a consent form. A recruitment graphic was also shared in pre-clerkship medical student social media groups. Interested parties were instructed to contact the researchers via email if interested in participating. In total, 16 participants were recruited. Five were in their first year of study, 10 in their second year, and one in their third year, at the time of the interview. The Western University’s Research Ethics Board approved this study.

### Data Collection

**Figure 1:**
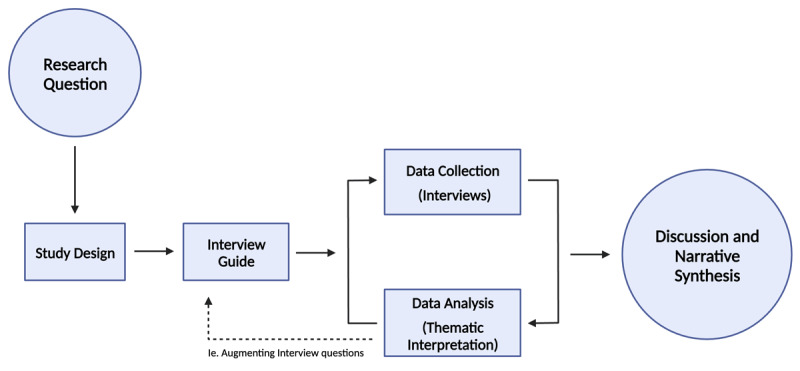
Iterative process of data analysis.

Virtual interviews were conducted via Zoom and were scheduled for 20 – 40 minutes, at the participant’s convenience. All interviews took place within the 2020–2021 and 2021–2022 academic years. Medical student researchers NO, AS, JJ, and MG facilitated the interviews to mitigate power differentials that might have existed with more senior researchers and to encourage students to speak freely. A semi-structured interview guide consisting of open-ended questions was used to allow the participant to express their experience, thoughts, and feelings about online learning more freely while also having a predetermined framework to gather more understanding of emerging themes (See Supplement A). All interviews were recorded, transcribed verbatim by the student researcher conducting the interview and subsequently anonymized. In keeping with the CGT approach, interview questions were refined throughout in an iterative process of data collection and analysis (Figure 1).

The diagram demonstrates the cyclical nature of data collection and analysis, which allowed for iterative modifications to the interview guide. Created with BioRender.com.

### Data Analysis

Interviews were independently analyzed by the student researcher who facilitated the given interview. For independent data analysis student researchers initially employed line-by-line open coding and created new codes as necessary for the first four interviews transcribed, with each student researcher having open-coded one transcribed interview. Through discussion among student researchers, we developed a set of focused codes – to eliminate overlap between similar codes – and applied these moving forward towards subsequent interviews. Student researchers independently coded transcribed interviews that they had conducted using the focused codes and occasionally created new codes to capture novel experiences/ ideas/ thoughts not captured by the focused codes. With regular discussion among the student researchers, as themes began to develop, students decided by consensus on refining the coding approach. After group discussions, transcripts were re-coded using the refined codes by at least two student researchers with the intention of corroborating our own analysis.

Student researchers regularly met for discussion and employed axial coding to examine and draw connections between existing codes. As analysis progressed this enabled the research team to move from categorizing data to interpreting data conceptually and constructing a model that explained the relationship between medical student well-being and online learning. During discussions student researchers employed the constant comparative approach customary in CGT [21] and decisions about the coding scheme and its evolution were made through consensus. Student researchers also met with CW at regular intervals to assist with the analysis and provide suggestions on refinements to the interview guide.

Consistent with the CGT approach, we recorded our developing analytic insights in a series of memos written after team discussions. To achieve theoretical saturation [22], we continually refined our interview guide to facilitate exploration of the full dimensions of the themes we identified. In addition, we engaged in theoretical sampling, broadening our initial recruitment plan to include students from multiple classes with varying combinations of virtual and in-person learning time to assess if our understanding of the developed thematic framework was applicable among heterogenous groups. This approach also enabled exploration of how students adapted to online learning over time. We ended data collection when we determined we had reached sufficiency which we defined as the point at which we had a logical conceptualization of the process we were studying without important gaps in our understanding.

Reflexivity was an ongoing process throughout the data collection and data analysis efforts. All student researchers who conducted interviews are medical students situated within the same learning environment as the participants. This established an environment where participants were able to easily explain the nuances of their experiences. However, as an interview team we were cognizant to not influence the participant statements in any way. Between interviews, researchers actively discussed strategies to communicate an understanding of the participant’s experience, without inferring if this experience was shared by the interviewer. During analysis, we purposefully voiced our own expectations of the data to allow other team members an opportunity to challenge how our own experiences might influence interpretation. Our research group also contained a clinician-scientist with a PhD in medical education (CW), whom we regularly consulted throughout the data collection and analysis.

## Results

**Figure 2:**
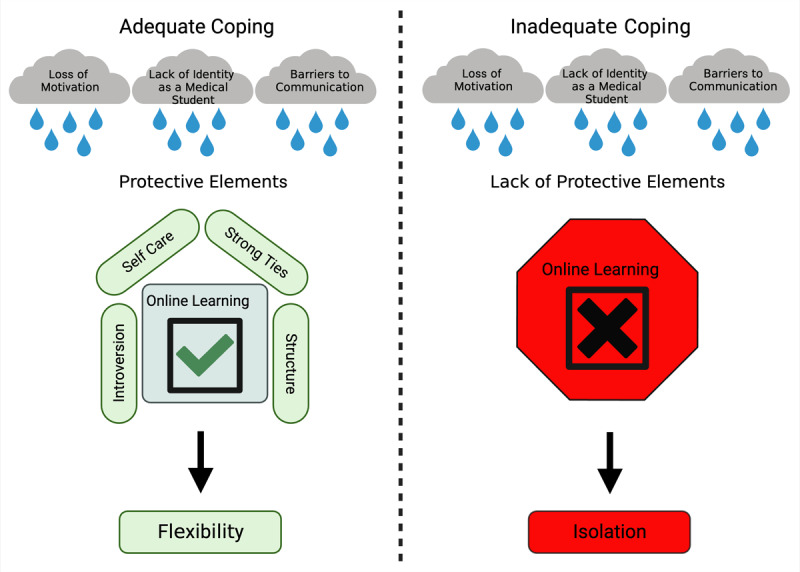
The impact of protective coping strategies on virtual learning experiences.

Data analysis revealed students were able to identify both positive and negative aspects of online learning that contributed to their mental well-being. Participants utilized various protective coping strategies to uphold the positives in the face of negative effects (Figure 2 Created with BioRender.com). The challenges to well-being that online learning produced coalesced around three major themes including barriers to 1) motivation, 2) communication, and 3) identity formation. We theorized that if students were able to balance the negative effects of online curriculum sufficiently with coping strategies, they were able to appreciate virtual learning for the flexibility it provided. However, if participants could not adequately apply protective measures, they experienced an isolated learning environment and ultimately a negative outlook.

The presence or absence of protective coping strategies shaped medical student outlook on virtual learning. Medical students who saw themselves as more introverted and utilized protective elements including the practice of self-care, strong ties to others and structured daily routines generally had a more positive outlook on virtual learning.

### Motivation

A predominant theme was that participants identified a decreased source of extrinsic motivation due to virtual learning. Students reported that they are “very motivated by [their] classmates and look forward to seeing them and learning alongside them” (P1). In contrast, students recognized virtual learning as devoid of human interaction as they “found it really frustrating to get up and just sit at my own desk every day, and not have experience with going to school and seeing my peers” (P9). In addition, “there’s less accountability with online learning” (P4) as there is reduced social pressure to be engaged with the content since “people can hide over zoom” (P3).

Achieving a sense of routine was beneficial for protecting against waning motivation: “I knew it’d be tough to separate working from home versus knowing when to cut it off and just relax…it just came down to being disciplined…These are the hours that I need to get my work done.” (P13). Self-care activities like exercise helped rejuvenate motivation: “Running is one thing that I find is a good outlet for me and has really helped me, but definitely there were times that I felt like oh my gosh I’m stuck in this one space and that really felt more difficult to focus” (P14). Being able to study in-person with classmates also provided some with accountability and external motivation: “I’ve been lucky throughout this whole year because I have my housemates that are also in our class but I can’t imagine for the students that are in our class that weren’t constantly around one or more classmates” (P6).

### Communication and connection

Participants reported barriers to communication with administration, peers, and faculty associated with virtual learning. While students recognized logistical issues were to be expected with the expedited transition to virtual learning and expressed sympathy towards administrative staff: “things became a little bit more difficult on the admins and there’s definitely a lot more questions from people” (P16). There was also persistent frustration with limited transparency: “it feels like there hasn’t been a lot of communication from the school” (P6). Frustrations also arose regarding reduced opportunities to build a learning community with peers: “we just all want to get out of that zoom as fast as possible. It’s not that we don’t want to get to know each other, but it’s like I’m tired of zoom, I can’t talk anymore, so we just log off. You don’t really get to know who you’re learning with” (P7). Ultimately this led to leading to weaker connections within the cohort, and similar difficulties were noted for connecting with faculty: “In terms of relationship building with physicians, that was really impacted” (P3). In online learning formats with faculty, students also felt more anonymous, “I’m just a name on the clipboard to them” (P11).

Protective elements mitigating these frustrations included pre-established connections, “I would say for me there wasn’t as big of an impact with my peers, because we were kind of the lucky class in that we had the first semester to form friend groups” (P16). Having an introverted personality type also seemed to exert protective effects: “I’m actually introverted…so I like having interactions in moderation” (P10). For these participants, online learning provided some relief from unnecessary interactions: “I could spend a lot more time doing what I wanted to do without the societal expectation to go and be social.” (P2).

### Identity

Many students reported the virtual curriculum altered the degree to which they identified with the school and as a medical student. “I have no connection to the school. I could be doing this from anywhere. I don’t really know any faculty. Except for like the research person I’m working with. I think it’s incredibly frustrating.” (P11). Although students recognized every medical school was going through a similar experience, we identified a common theme where the virtual platform and clinical restrictions imposed by the pandemic made students feel “different from previous medical students…These people would have had more in person sessions and they would have done all these clinical skills and all these clubs and volunteering, and I couldn’t relate to any of that because I didn’t have that opportunity.” (P7). Others were seemingly more affected by the changing curriculum delivery, reporting a difficult time identifying as a medical student: “In terms of my identity as a medical student? Sometimes I don’t really feel like one” (P3).

Ultimately this lack of connection resulted in some participants reporting a lack of confidence when sharing this achievement with friends and family:

“I think it changes my confidence when I tell people who aren’t in medicine that I’m a medical student… when you tell someone who’s not in medical school like your friends or your family, they are so proud and they talk about how hard it is or how hard it must be and all these things, and on the inside I often think, I don’t really feel like a medical student though… And that might just be a function of being a pre-clerk but I think the online learning aspect has to do with it” (P8).

Additionally, some students worried about how their clinical abilities would be perceived by future preceptors: “Will the doctors be like, oh, the class of 2024, like, oh, the class that did school online”… I don’t know, I hope not.” (P3).

Strong connections with other students appeared to mitigate the lack of identity concerns. “I’m lucky because I live with a couple of classmates. So that’s kept that human connection side of it and seeing other people that are in the same boat. And we talked about school together. So I still feel like I had a little bit of normalcy there” (P6).

### Flexibility or isolation

Many students noted appreciation for the inherent flexibility of how online education is “adaptable to each person’s learning style” (P16). However, some participants – particularly those with minimal protective factors in place – reported overall negative mental health effects related to virtual learning. Participants described mood disturbances: “I can feel I’m getting more emotionally labile, and I feel like I’m on the verge of a breakdown constantly now. Just in front of the computer all day.” (P9), and depression symptoms: “You have so much to learn and you don’t have an outlet to de-stress…I’m just constantly in this fatigued, tired, sad state” (P7). Ultimately, these students articulated that their medical school experience: “feels very isolating” (P11), and virtual learning led to a reduction in class community. “Being in person forces you to interact with everyone on a more regular basis to keep that broader community sense.” (P1).

## Discussion

The uncertainty associated with COVID-19 related social restrictions and subsequent last-minute curricular changes meant that students did not have time to prepare for the circumstances related to virtual education and had to adapt extremely quickly. Once they adapted, however, they seemed to appreciate certain aspects of the virtual curriculum and its associated freedoms. Recent studies have found medical students are satisfied with many aspects of virtual learning [23,24]. Our analysis suggests, however, that the impact of online learning on medical students is a more complex matter than satisfaction with the virtual approach to education.

When virtual learning was implemented, many students reported a significant drop in motivation. However, they were concurrently presented with a surge in freedom and flexibility. It is possible the gradual benefit of this increased flexibility impacted the students’ motivational state. In other words, as they became more familiar with the curricular landscape, they found ways to maintain their wellness and their motivation returned. This raises the questions; how long do we need to wait before assessing the impact of curricular changes or the introduction of new learning modalities on student well-being? How can we facilitate students’ adjustment to these changes? While answers to these questions are important avenues for future research, our work reminds us of the range of impacts curricular change can have on well-being and offers targets for us to enable better learner preparation for that change. While some of these targets are well-known already (i.e. the need for clear communication), our work highlights the central importance of a previously-overlooked issue: the impact of virtual learning on professional identity formation.

Professional identity formation is the process through which trainees evolve from members of the lay public to working professionals [25]. It has been established that medical students, residents and attending physicians have distinct professional identities [26] and it is known that the development of one’s professional identity becomes frustrating and stressful to trainees when dissonance occurs between one’s existing identity and the requirements of their surroundings [27].

So how do we support medical students in the development of their professional identity? The current literature establishes two key factors for fostering professional identity formation 1) role models and mentors and 2) experiential learning both in and out of clinical settings [28,29]. Self-reflection, ideally guided by a mentor is also known to aid this process [30]. It is important to consider the effects of virtual learning on these factors. Students reported virtual learning limited their access to faculty. This may limit mentorship accessibility and in turn may harm the development of professional identity. Additionally, virtual learning may limit opportunities for experiential learning both in and out of the clinical setting, further harming the development of trainees’ professional identities.

It stands to reason that a trainee must achieve the professional identity within each step of the medical training process, to confidently take on increasing levels of responsibility. In fact, feeling competent has been reported to be critical in this process [28]. Many students expressed concerns regarding being thought of as less competent since they were a trainee who had received virtual education with limited access to in-person training. If online learning impairs a medical student’s ability to identify as a medical student and build the requisite confidence to excel in this role, this may in turn cascade to later stages in their training.

### Implications for medical schools

Many students offered opinions on which components of their training should remain virtual and which should return to in-person moving forward. While small group learning and didactic lecture-style content had pros and cons for both forms of delivery, there was a strong desire for clinical skills and anatomy to be delivered in-person. Many students resonated with hands-on learning being the optimal way to learn these skills and expressed concern with these components when virtually delivered. Ensuring clinical skills are taught in person would enhance opportunities for students to interact with faculty and therefore find mentors and engage in experiential learning, two key factors known to be critical in the development of professional identity [31]. Additionally, since isolation was an overarching negative theme identified in this study, schools should seek to capitalize on and generate opportunities to encourage peer-peer interaction beyond the classroom.

### Study Limitations

We acknowledge important limitations exist with this study including sample size (although interviewing to saturation was completed to mitigate this issue) and an inability to completely separate the effects of the pandemic and the effects of virtual learning on medical student wellbeing. Additionally, the COVID-19 pandemic greatly impacted the mental health of people worldwide [32]. While not occurring in a vacuum during the pandemic, the switch to online learning and its contributions to changes in medical student wellbeing, especially during such a time as a global pandemic, should be carefully considered. However, this study offers insight into the medical student experience across different timepoints during the COVID-19 pandemic with virtual learning. While most published research exploring similar populations during this time has been quantitative and/or hypothesis driven [20], our qualitative approach facilitates a deeper conceptual understanding of how medical students experience and adapt to virtual learning.

## Conclusion

The rapid shift towards virtual learning during the COVID-19 pandemic was a dramatic change to medical education. There were both positive and negative effects on medical student well-being associated with virtual learning. The limitations surrounding communicating with peers and mentors in a virtual world negatively impacted students’ motivation, enhanced feelings of isolation, and disrupted their identity as medical students. The consequences of interrupting or even impeding the development of professional identity at this critical timepoint in trainee development may in fact be still to come, and should be targets of future research. On the other hand, we also observed that students, once given more time to adapt, often came to value the flexibility afforded by online learning. Ultimately, when incorporating virtual learning into the medical school experience, medical schools should optimize existing and generate new ways for students to interact with each other and faculty to strengthen medical student identity and combat feelings of social isolation.

## Additional File

The additional file for this article can be found as follows:

10.5334/pme.1184.s1 Supplement A. Interview Guide.
