# Supplementary material for: Examining the Effect of Virtual Learning on Canadian Pre-Clerkship Medical Student Well-Being During the COVID-19 Pandemic
Source: Perspect Med Educ. 2023 Nov 2;12(1):488–96. doi: 10.5334/pme.1184 (PMC10624142; doi:10.5334/pme.1184)
Supplement: Supplement A. — Interview Guide. [file pme-12-1-1184-s1.pdf]

Supplement A - Interview Guide

Please note the following questions are not meant to be an inclusive list of possible questions to be asked during the interview, moreover not every question will be asked. The interviewer can use their judgement to formulate and pose new questions to the participant depending on the direction/topic of the interview. This list of questions will serve as a guide for the interviewer. This guide may also be updated iteratively as insights emerge in accordance with a grounded theory methodological approach.

Potential Online-Learning Subtopics:

- Lack of preparedness for clerkship (substandard medical education),
- Isolation and loneliness (lacking connection to peers and the larger class)
- Fear of missing out on “medical school life”
- Disruption of a routine
- Access to technology (stable WiFi, quiet home environment)

Guiding Questions:

1. Can you tell me about your experience with online learning in medical school so far? How has it been?
    1. What part of online learning is the most distressing?
    2. What part of online learning do you enjoy the most?
2. How did you first feel about the online curriculum when it was introduced in September?
    1. Have you become accustomed to the online curriculum? Are you used to it, prefer it, or are just tolerating it?
    2. What part of online learning became worse over time? What part became better over time?
3. Since our curriculum is predominantly online, has that affected your relationships between your peers, classmates?
    1. How has that made you feel / impacted your mental health? What goes through your mind?
    2. Do you fear that you’re missing out?
    3. What would you have liked to happen ideally?
    4. How do you cope with this (loneliness)?
4. Do you find yourself sitting at your computer all day, more than usual?
    1. How does that make you feel? How does this influence your behaviour?
    2. How do you cope with this?
5. How do you feel about the possibility of returning back to an in-person curriculum?
    1. What are you worried about?
    2. What are you excited about?
6. How does online learning fit in with your daily routine?
    1. Has online learning disrupted a routine you had or one you envisioned for yourself?
    2. Is online medical learning a better fit for your schedule? Does it keep you more disciplined?

    3. Has this affected your motivation in any way?
7. Do you have all the resources necessary for online learning, are there ever any barriers that you’ve experienced with online learning?
    1. How has that affected you? How has it made you feel?
    2. How do you deal with those feelings? How do you deal with not having proper access?
8. Do you believe you’re getting the standard of medical education that you would receive in other years, if there was an in-person curriculum?
    1. How do you think this will affect your readiness for clerkship, if at all? How do you think it might affect your readiness for qualifying exams and residency?
    2. How does this make you feel? Does this make you feel anxious, hopeless, etc.?
